# Author Correction: A human progeria-associated BAF-1 mutation modulates gene expression and accelerates aging in *C. elegans*

**DOI:** 10.1038/s44318-025-00432-1

**Published:** 2025-04-11

**Authors:** Raquel Romero-Bueno, Adrián Fragoso-Luna, Cristina Ayuso, Nina Mellmann, Alan Kavsek, Christian G Riedel, Jordan D Ward, Peter Askjaer

**Affiliations:** 1 Andalusian Centre for Developmental Biology, Consejo Superior de Investigaciones Científicas (CSIC), Universidad Pablo de Olavide, Junta de Andalucía, Carretera de Utrera, km 1, 41013 Sevilla, Spain; 2 Department of Biosciences and Nutrition, Karolinska Institutet, Huddinge, 14157 Sweden; 3 Department of Molecular, Cell, and Developmental Biology, University of California-Santa Cruz, Santa Cruz, CA 95064 USA

## Abstract

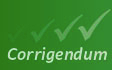

**Correction to:** *The EMBO Journal* (2024) 43: 5718–5746. 10.1038/s44318-024-00261-8 | Published online 4 October 2024

**Figure 2B is withdrawn and replaced**.

The authors contacted the journal after identifying an error in Figure 2B and D of the published paper. After reviewing the corrected figure provided by the authors, the journal agrees to withdraw and replace Figure 2B.

Author statement:

Figure 2B (top, Day 1) was inadvertently duplicated from Figure 2D (top, Day 1). The corresponding data in Table EV1A,B remain correct.

**Figure 2:**
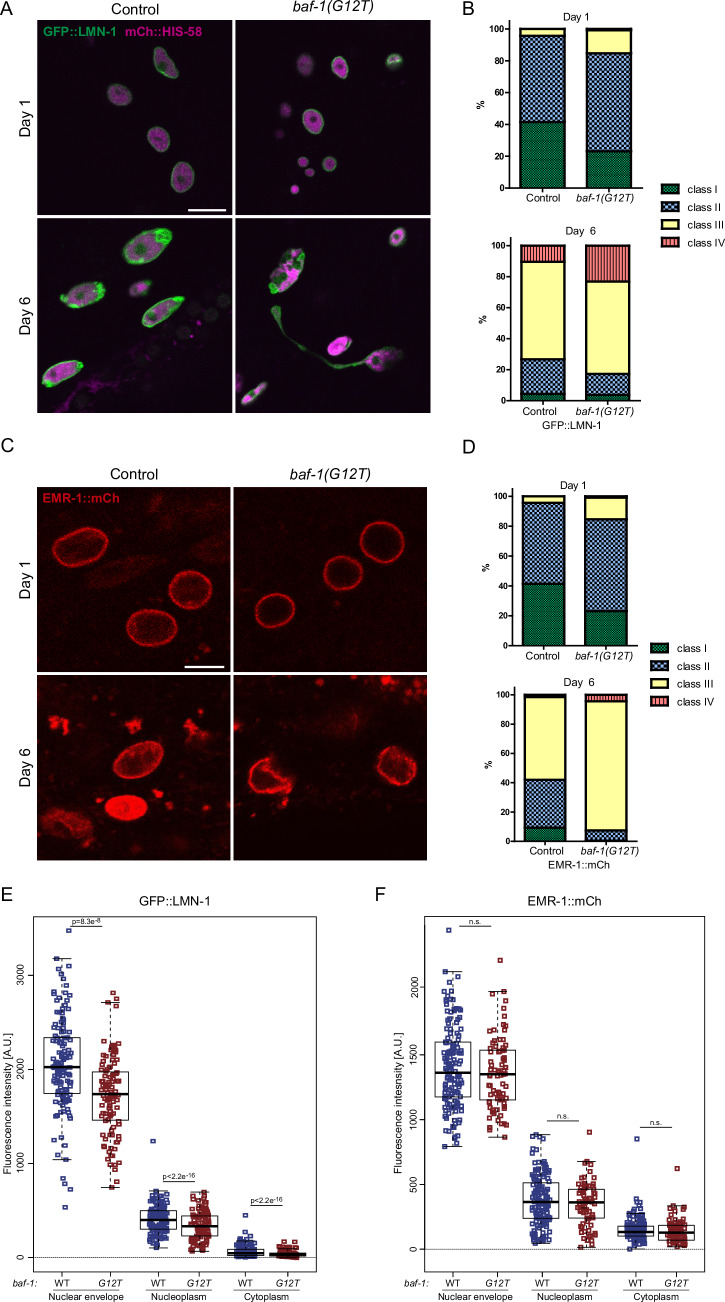
Original

**Figure 2:**
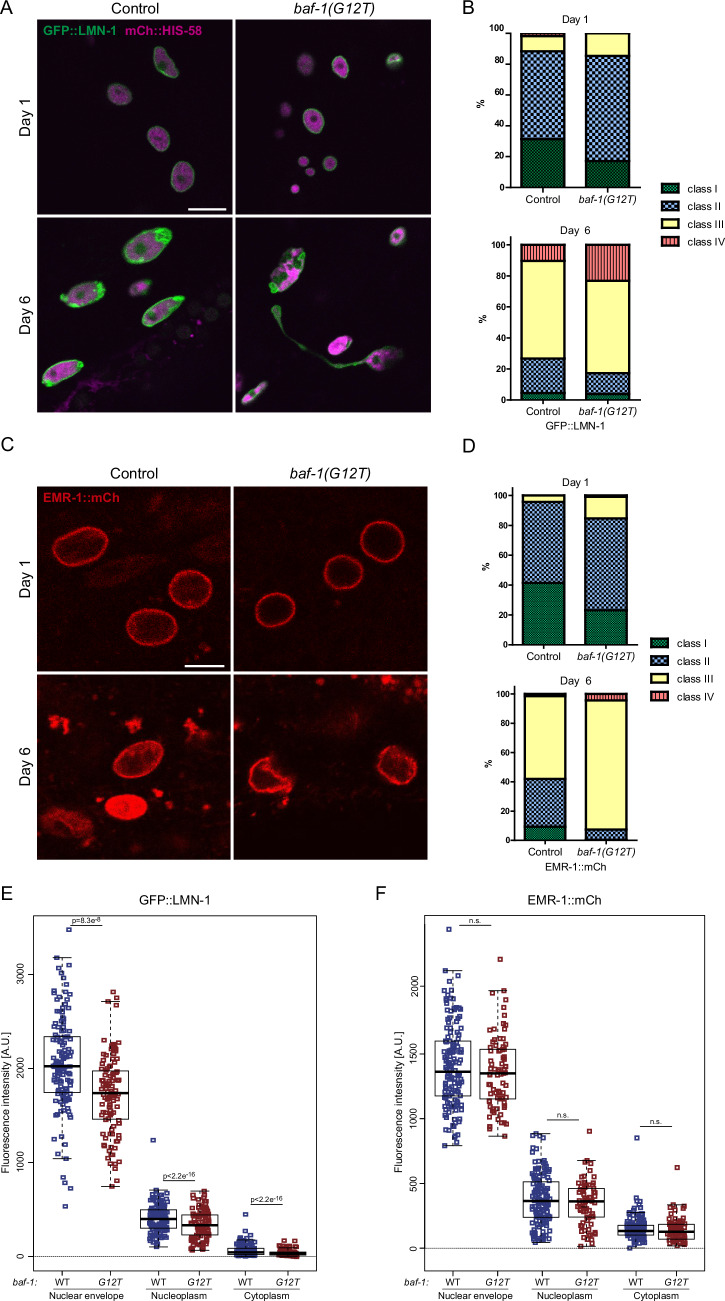
Corrected

We have provided the corrected Figure 2 for replacement. This correction does not affect the integrity of the results or conclusions presented in the study.

All authors agree to this correction.

