# Supplementary material for: A human progeria-associated BAF-1 mutation modulates gene expression and accelerates aging in C. elegans
Source: EMBO J. 2024 Oct 4;43(22):18. doi: 10.1038/s44318-024-00261-8 (PMC11574047; doi:10.1038/s44318-024-00261-8)
Supplement: Supplementary file 1 — Table EV1 [file 44318_2024_261_MOESM1_ESM.pdf]

Table EV1A

GFP::LMN-1						
	Day 1		Day 6		Day 8	
Class	WT	*baf-1(G12T)*	WT	*baf-1(G12T)*	WT	*baf-1(G12T)*
I	35	21	9	9	1	8
II	64	84	45	32	47	45
III	11	18	127	141	64	114
IV	2	0	21	55	9	40
Total	112	123	202	237	121	207
Fisher's Exact Test p-values comparing WT and baf-1(G12T)						
Class I vs II+III+IV	0.029					
Class I+II vs III+IV			0.020		0.0093	
Class I+II+III vs IV			0.00038		0.0036	

Table EV1B

EMR-1::mCherry						
	Day 1		Day 6		Day 8	
Class	WT	*baf-1(G12T)*	WT	*baf-1(G12T)*	WT	*baf-1(G12T)*
I	29	27	25	1	0	5
II	38	72	87	16	43	19
III	3	17	151	202	219	177
IV	0	1	4	10	9	21
Total	70	117	267	229	271	222
Fisher's Exact Test p-values comparing WT and baf-1(G12T)						
Class I vs II+III+IV	0.013					
Class I+II vs III+IV			< 2.20E-16		0.11	
Class I+II+III vs IV			0.062		2.53E-06	

Table EV1C

Combined						
	Day 1		Day 6		Day 8	
Class	WT	*baf-1(G12T)*	WT	*baf-1(G12T)*	WT	*baf-1(G12T)*
I	64	48	34	10	1	13
II	102	156	132	48	90	64
III	14	35	278	343	283	291
IV	2	1	25	65	18	61
Total	182	240	469	466	392	429
Fisher's Exact Test p-values comparing WT and baf-1(G12T)						
Class I vs II+III+IV	0.00055					
Class I+II vs III+IV			< 2.20E-16		0.069	
Class I+II+III vs IV			7.21E-06		2.53E-06	

Classification of hypodermal nuclei expressing GFP::LMN-1 (A) or EMR-1::mCherry (B) according to their morphology (see Material and Methods). Combined data from (A) and (B) are represented in (C).
