# Supplementary material for: A human progeria-associated BAF-1 mutation modulates gene expression and accelerates aging in C. elegans
Source: EMBO J. 2024 Oct 4;43(22):18. doi: 10.1038/s44318-024-00261-8 (PMC11574047; doi:10.1038/s44318-024-00261-8)
Supplement: Supplementary file 2 — Table EV2 [file 44318_2024_261_MOESM2_ESM.pdf]

Table EV2

		Arm	Center	Ratio arm/center	p-value[a]
	All autosome bins	5133	3125	1.6	
Hypodermis	Enriched WT[b]	358	52	6.9	< 2.20E-16
	Enriched G12T[c]	228	250	0.9	5.03E-10
Intestine	Enriched WT[b]	320	266	1.2	0.00036
	Enriched G12T[c]	351	246	1.4	0.106

[a] Fisher's Exact Test p-values

[b] Bin enriched for WT (FDR <0.05; fold change >2) and that fulfil log2(Dam::BAF-1/GFP::Dam) - log2(Dam::BAF-1(G12T)/GFP::Dam) >0.58

[c] Bin enriched for G12T (FDR <0.05; fold change >2) and that fulfil log2(Dam::BAF-1(G12T)/GFP::Dam) - log2(Dam::BAF-1/GFP::Dam) >0.58
