# Supplementary material for: A human progeria-associated BAF-1 mutation modulates gene expression and accelerates aging in C. elegans
Source: EMBO J. 2024 Oct 4;43(22):18. doi: 10.1038/s44318-024-00261-8 (PMC11574047; doi:10.1038/s44318-024-00261-8)
Supplement: Supplementary file 3 — Table EV3 [file 44318_2024_261_MOESM3_ESM.pdf]

Table EV3

Strain	Description	Genotype	Method	Reference
BN148	Ubiquitous expression of EMR-1::mCh from integrated single-copy transgene; rescues *emr-1(gk119)* mutation	*emr-1(gk119) I; bqSi143[emr-1p::emr-1::mCh] II*	MosSCI	(Morales-Martinez et al. 2015)
BN189	Ubiquitous expression of mCh::HIS-58 from integrated single-copy transgene	*bqSi189[lmn-1p::mCh::his-58)] II*	MosSCI	(Gomez-Saldivar et al. 2016)
BN448	For tissue-specific control DamID after crossing to FLP driver	*bqSi447[hsp16.41p::FRT::mCh::his-58::FRT::gfp::dam] II*	MosSCI	(Cabianca et al. 2019))
BN536	For tissue-specific BAF-1 DamID after crossing to FLP driver	*bqSi536[hsp16.41p>mCh::his-58>dam::baf-1] II*	MosSCI	This study
BN548	Expression of FLP in hypodermis	*bqSi548[dpy-7p::FLP] IV*	MosSCI	(Munoz-Jimenez et al. 2017)
BN561	Control DamID in hypodermis	*bqSi447[hsp16.41p::FRT::mCh::his-58::FRT::gfp::dam] II; bqSi548[dpy-7p::FLP] IV*	MosSCI	(Fragoso-Luna et al. 2023)
BN580	GFP knock-in into 5'-end of *baf-1* CDS; Frt sites in 1st and 2nd intron of GFP; designed for tissue-specific gene knockout	*baf-1(bq12[g>f>p::baf-1]) III*	CRISPR	(Munoz-Jimenez et al. 2017)
BN581	mCh knock-in into 5'-end of *baf-1* CDS	*baf-1(bq13[mCh::baf-1]) III*	CRISPR	(Fragoso-Luna et al. 2023)
BN599	Ubiquitous expression of mCh::HIS-58 and GFP::BAF-1	*bqSi189[lmn-1p::mCh::his-58] II; baf-1(bq12[g>f>p::baf-1]) III*	BN189 crossed with BN580	This study
BN777	For tissue-specific BAF-1(G12T) DamID after crossing to FLP driver and *baf-1(G12T)*	*bqSi777[hsp16.41p>mCh::his-58>dam::baf-1(G12T)] II*	MosSCI	This study
BN808	*baf-1(G12T)* mutant	*baf-1(bq19[G12T]) III*	PMX375 crossed to N2 6 times	This study
BN868	*baf-1(G12T)* mutant; uncoordinated	*baf-1(bq19[G12T]) unc-119(ed3) III*	BN808 crossed with HT1593	This study
BN869	Ubiquitous expression of mCh::HIS-58 and GFP::LMN-1	*lmn-1(yc32[gfp::lmn-1]) I; bqSi189[lmn-1p::mCh::his-58] II*	BN189 crossed with UD484	This study
BN870	*baf-1(G12T)* mutant with ubiquitous expression of mCh::HIS-58 and heterozygous expression of GFP::LMN-1	*lmn-1(yc32[gfp::lmn-1])/+ I; bqSi189[lmn-1p::mCh::his-58)] II; baf-1(bq19[G12T]) III*	BN808 crossed with BN189 and UD484	This study
BN922	*baf-1(G12T)* mutant with ubiquitous expression of EMR-1::mCh	*emr-1(gk119) I; bqSi143[emr-1p::emr-1::mCh] II; baf-1(bq19[G12T]) III*	BN148 crossed with BN808	This study
BN998	Expression of FLP in intestine	*bqSi997[nhx-2p::FLP::SL2::mNG] IV*	MosSCI	(Fragoso-Luna et al. 2023)

BN1007	GFP knock-in into 5'-end of *baf-1(G12T)* CDS; Frt sites in 1st and 2nd intron of GFP; balanced w/ mT1	*+/mT1 [umnIs34] II; baf-1(bq24[G>F>P::baf-1[G12T]])/mT1 [dpy-10(e128)] III*	CRISPR	This study
BN1024	mCh knock-in into 3'-end of *emr-1* CDS; also Frt site in 5'-end of *emr-1* CDS	*emr-1(bq34[emr-1::mCh]) I*	CRISPR	This study
BN1037	mCh knock-in into 3'-end of *emr-1* CDS; GFP knock-in into 5'-end of *lmn-1* CDS	*emr-1(bq34[emr-1::mCh]) lmn-1(yc32[gfp::lmn-1]) I*	BN1024 crossed with UD484	This study
BN1047	BAF-1 DamID in hypodermis	*bqSi536[hsp16.41p>mCh::his-58>dam::baf-1] II; bqSi548[dpy-7p::FLP] IV*	BN536 crossed with BN548	This study
BN1048	Control DamID in hypodermis of *baf-1(G12T)*	*bqSi447[hsp16.41p::FRT::mCh::his-58::FRT::gfp::dam] II; baf-1(bq19[G12T]) III; bqSi548[dpy-7p::FLP] IV*	BN561 crossed with BN808	This study
BN1050	BAF-1(G12T) DamID in hypodermis	*bqSi777[hsp16.41p>mCh::his-58>dam::baf-1(G12T)] II; baf-1(bq19[G12T]) III; bqSi548[dpy-7p::FLP] IV*	BN548 crossed with BN777 and BN808	This study
BN1051	Control DamID in intestine	*bqSi447[hsp16.41p>mCh::his-58>gfp::dam] II; bqSi997[nhx-2p::FLP::SL2::mNG] IV*	BN448 crossed with BN998	This study
BN1052	BAF-1 DamID in intestine	*bqSi536[hsp16.41p>mCh::his-58>dam::baf-1] II; bqSi997[nhx-2p::FLP::SL2::mNG] IV*	BN536 crossed with BN998	This study
BN1053	Control DamID in intestine of *baf-1(G12T)*	*bqSi447[hsp16.41p>mCh::his-58>gfp::dam] II; baf-1(bq19[G12T]) III; bqSi997[nhx-2p::FLP::SL2::mNG] IV*	BN808 crossed with BN1051	This study
BN1054	BAF-1(G12T) DamID in intestine	*bqSi777[hsp16.41p>mCh::his-58>dam::baf-1(G12T)] II; baf-1(bq19[G12T]) III; bqSi997[nhx-2p::FLP::SL2::mNG] IV*	BN777 crossed with BN808 and BN998	This study
BN1150	mCh knock-in into 3'-end of emr-1 CDS and GFP knock-in into 5'-end of lmn-1 CDS	*emr-1(bq34[emr-1::mCh]) lmn-1(yc32[gfp::lmn-1]) I*	BN1024 crossed with UD484 and then with N2 4 times	This study
BN1188	GFP knock-in into 5'-end of *baf-1(G12T)* CDS; ubiquitous expression of mCh::HIS-58; balanced w/ mT1	*bqSi189[lmn-1p::mCherry::his-58]/mT1 [umnIs34] II; baf-1(bq24[G>F>P::baf-1[G12T]])/mT1 [dpy-10(e128)] III*	BN189 crossed with BN1007	This study
BN1337	Balanced *baf-1(G12T)* mutant with ubiquitous expression of mCh::HIS-58 and heterozygous expression of GFP::LMN-1	*lmn-1(yc32[gfp::lmn-1]) I/hT2 (I;III); bqSi189[lmn-1p::mCherry::his-58] II; baf-1(bq19[G12T]) III/hT2 (I;III)*	BN870 crossed with hT2 strain	This study

BN1349	mCh knock-in into 5'-end of *baf-1* CDS; temperature-sensitive; at 25C animals lack germ line	*glp-4(bn2) I; baf-1(bq13[mCh::baf-1]) III*	BN581 crossed with SS104	This study
BN1350	*baf-1(G12T)* mutant; temperature-sensitive; at 25C animals lack germ line	*glp-4(bn2) I; baf-1(bq19[G12T]) III*	BN808 crossed with BN1349	This study
BN1375	Temperature-sensitive; at 25C animals lack germline	*glp-4(bn2) I*	BN808 crossed with BN1349	This study
BN1412	Tissue-specific RPB-6 DamID	*bqSi1411[hsp16.41p::FRT::mCh::his-58::FRT::dam::rpb-6] II; bqSi577[myo-2p::GFP)] IV*	MosSCI	(Fragoso-Luna et al. 2023)
BN1414	RPB-6 DamID in hypodermis	*bqSi1411[hsp16.41p::FRT::mCh::his-58::FRT::dam::rpb-6] II; bqSi548[dpy-7p::FLP] IV*	MosSCI	(Fragoso-Luna et al. 2023)
BN1415	RPB-6 DamID in intestine	*bqSi1411[hsp16.41p::FRT::mCh::his-58::FRT::dam::rpb-6] II; bqSi997[nhx-2p::FLP::SL2::mNG] IV*	BN998 crossed with BN1412	This study
BN1416	RPB-6 DamID in hypodermis in *baf-1(G12T)*	*bqSi1411[hsp16.41p::FRT::mCh::his-58::FRT::dam::rpb-6] II; baf-1(bq19[G12T]) III; bqSi548[dpy-7p::FLP] IV*	BN808 crossed with BN1414	This study
BN1417	RPB-6 DamID in intestine in *baf-1(G12T)*	*bqSi1411[hsp16.41p::FRT::mCh::his-58::FRT::dam::rpb-6] II; baf-1(bq19[G12T]) III; bqSi997[nhx-2p::FLP::SL2::mNG)] IV*	BN808 crossed with BN1415	This study
CB4108	Feminized hermaphrodites unable to producing sperm	*fog-2(q71) V*		(Katju et al. 2008)
EG4322	MosSCI targeting strain	*ttTi5605 II; unc-119(ed9) III*		(Katju et al. 2008)
HT1593	Uncoordinated	*unc-119(ed3) III*		Dickinson et al. 2013)
N2	*C. elegans* var Bristol			CGC
PMX375	*baf-1(G12T)* mutant	*baf-1(bq19[G12T]) III*	CRISPR	This study
SS104	Temperature-sensitive; at 25C animals lack germline	*glp-4(bn2) I*		(Beanan & Strome, 1992)
UD484	GFP knock-in into 5'-end of *lmn-1* CDS	*lmn-1(yc32[gfp::lmn-1]) I*	CRISPR	(Bone et al. 2016)
UV117	GFP knock-in into 5'-end of *lmn-1* CDS	*lmn-1(jf98[lmn-1::GFP]) I*	CRISPR	(Link et al. 2018)

Strains used in this study

References:

Beanan MJ, Strome S. 1992. Characterization of a germ-line proliferation mutation in C. elegans. *Development* **116**: 755-766.

Bone CR, Chang YT, Cain NE, Murphy SP, Starr DA. 2016. Nuclei migrate through constricted spaces using microtubule motors and actin networks in C. elegans hypodermal cells. *Development* **143**: 4193-4202.

Cabianca DS, Munoz-Jimenez C, Kalck V, Gaidatzis D, Padeken J, Seeber A, Askjaer P, Gasser SM. 2019. Active chromatin marks drive spatial sequestration of heterochromatin in C. elegans nuclei. *Nature* **569**: 734-739.

Dickinson DJ, Ward JD, Reiner DJ, Goldstein B. 2013. Engineering the Caenorhabditis elegans genome using Cas9-triggered homologous recombination. *Nat Methods* **10**: 1028-1034.

Fragoso-Luna A, Romero-Bueno R, Eibl M, Ayuso C, Munoz-Jimenez C, Benes V, Cases I, Askjaer P. 2023. Expanded FLP toolbox for spatiotemporal protein degradation and transcriptomic profiling in Caenorhabditis elegans. *Genetics* **223**.

Frøkjær-Jensen C, Davis MW, Hopkins CE, Newman BJ, Thummel JM, Olesen SP, Grunnet M, Jorgensen EM. 2008. Single-copy insertion of transgenes in Caenorhabditis elegans. *Nat Genet* **40**: 1375-1383.

Gomez-Saldivar G, Fernandez A, Hirano Y, Mauro M, Lai A, Ayuso C, Haraguchi T, Hiraoka Y, Piano F, Askjaer P. 2016. Identification of Conserved MEL-28/ELYS Domains with Essential Roles in Nuclear Assembly and Chromosome Segregation. *PLoS Genet* **12**: e1006131.

Katju V, LaBeau EM, Lipinski KJ, Bergthorsson U. 2008. Sex change by gene conversion in a Caenorhabditis elegans fog-2 mutant. *Genetics* **180**: 669-672.

Link J, Paouneskou D, Velkova M, Daryabeigi A, Laos T, Labella S, Barroso C, Pacheco Pinol S, Montoya A, Kramer H et al. 2018. Transient and Partial Nuclear Lamina Disruption Promotes Chromosome Movement in Early Meiotic Prophase. *Dev Cell* **45**: 212-225 e217.

Morales-Martinez A, Dobrzynska A, Askjaer P. 2015. Inner nuclear membrane protein LEM-2 is required for correct nuclear separation and morphology in C. elegans. *J Cell Sci* **128**: 1090-1096.

Munoz-Jimenez C, Ayuso C, Dobrzynska A, Torres-Mendez A, Ruiz PC, Askjaer P. 2017. An Efficient FLP-Based Toolkit for Spatiotemporal Control of Gene Expression in Caenorhabditis elegans. *Genetics* **206**: 1763-1778.
