# Supplementary material for: A human progeria-associated BAF-1 mutation modulates gene expression and accelerates aging in C. elegans
Source: EMBO J. 2024 Oct 4;43(22):18. doi: 10.1038/s44318-024-00261-8 (PMC11574047; doi:10.1038/s44318-024-00261-8)
Supplement: Supplementary file 4 — Table EV4 [file 44318_2024_261_MOESM4_ESM.pdf]

Table EV4

Primer ID	Description	Sequence
B633	generation of pBN180	5'-AACGTCGTGACTGGGAAAAC
B634	generation of pBN180	5'-GGTGCCAACTTTTCTATACAAAG
B635	universal sgRNA primer	5'-CAAGACATCTCGCAATAGGAG
B724	dpy-10; sgRNA sequence in capitals	5'-CTACCATAGGCACCACGAGgttttagagctagaaatagcaagt
B725	dpy-10; repair template	5'-CACTTGAACTTCAATACGGCAAGATGAGAATGACTGGAAACCGTACCGCATGCGGTGCCTATGGTAGCGGAGCTTCACATGGCTTCAGACCAACAGCCTAT
B780	generation of pBN215	5'-aaacagcatagcaagtttAAATAAGGCTAGTCCGTTATC
B781	generation of pBN215	5'-ccagcatagctcttaaacCAAGACATCTCGCAATAG
B825	emr-1 3'-end; sgRNA sequence in capitals	5'-CGTGGCCGAGACGAATCCGGgtttaagagctatgctggaaac
B838	emr-1 5'-end; sgRNA sequence in capitals	5'-AGACGGAAGCATTAACAGTGgtttaagagctatgctggaaac
B840	emr-1::mCh gBlock w/ Frt sites	5'-TGGGCCAATTGTGGCGACGACCCGCAAGCTCTACGAGAAGAAGCTTATCAAGTTATCAGAtGGAAGCATTAACAgtgagCatttgaatttaaattatttattaatttctGAAGTTCCTATTCTCTAGAAAGTATAGGAACTTCaaataacatttttaaagttttcgaacctctaatttccagaTCAATCAAATCTCAACGACTCTCAATTCAACGAGGATTCATTGATCATCAGCTCGTCACCGAAGAAATCACCGCCACAACGAGTTTTCCAGAACGTGTCAGCTGCAACAGCGGCAGCTACTACCTCTCCCGAATCGGACAGCGACGATTGCGAGGAGTCGATGCGATACTTGACGGAAGAGGAAATGGCCGCCGATCGGGCATCGGCTCGTCAAGCTCAGAGCAACAAAGGAGGATTCTTGGGAAGCACGgtgagtttttcgccctttttctggcaataaaaacttattttctattttccagATCACATTCACAATTCTCTTCGTCTTCATCGCCGTCTTCGCCTACTTCTTGATCGAGAACGCCGAGCAGTTGAAGCTCGTGGCCGAGACGAATCCaGAaGATACTATTtgtacaGTCTCAAAGGGTGAAGAAGATAACATGGCAATTATTAAAGAGTTTATGCGTTTCAAGGTGCATATGGAGGGATCTGTCAATGGGCATGAGTTTGAAATTGAAGGTGAAGGAGAAGGCCGACCATATGAGGGAACACAAACCGCAAAACTAAAGgtaagtttaaacatatatatGAAGTTCCTATTCTCTAGAAAGTATAGGAACTTCactaactaaccctgattatttaaattttcagGTAACTAAAGGCGGACCATTACCATTCGCCTGGGACATCCTCTCTCCACAGTTCATGTATGGAAGTAAAGCTTATGTTAAACATCCGGCAGATATACCAGATTATTTGAAACTTTCATTCCCGGAGGGTTTTAAGTGGGAACGCGTAATGA
B914	AdRt	5'-CTAATACGACTCACTATAGGGCAGCGTGGTCGCGGCCGAGGA
B919	AdRb	5'-TCCTCGGCCGCG
B920	AdR	5'-NNNNGTGGTCGCGGCCGAGGATC
B925	baf-1 5'-end	5'-caagatctATGTCGACTTCTGTTAAGCATCG
B926	baf-1 3'-end	5'-tagctagcTTACATGAACTGATCTGCCC
B1436	AdR4N	5'-NNNNGTCCTCGCGGCCGAGGATC
B1437	AdR5N	5'-NNNNNGTCCTCGCGGCCGAGGATC
B1438	AdR6N	5'-NNNNNNGTCCTCGCGGCCGAGGATC
B1103	pmp-3 qRT-PCR	5'-TGGCCGGATGATGGTGTCGC
B1104	pmp-3 qRT-PCR	ACGAACAATGCCAAAGGCCAGC
B1105	tba-1 qRT-PCR	TCAACACTGCCATCGCCGCC
B1106	tba-1 qRT-PCR	TCCAAGCGAGACCAGGCTTCAG
B1107	Y45F10D.4 qRT-PCR	CGAGAACCCGCGAAATGTCGGA
B1108	Y45F10D.4 qRT-PCR	CGGTTGCCAGGGAAGATGAGGC
B1603	baf-1 qRT-PCR	ATGCAGGCTTCGATAAAGCCTAC
B1604	baf-1 qRT-PCR	CAGCCGTCTCTTTCAGCCAT
B1605	emr-1 qRT-PCR	GCATTAACAATCAATCAAATCTC
B1606	emr-1 qRT-PCR	AAAACTCGTTGTGGCGGTGA
B1607	lmn-1 qRT-PCR	TGGTGGTGGAGAATGATGATCTC
B1608	lmn-1 qRT-PCR	CGGCTGTTCGGAGAAGAGTT

Primers used in this study
