# Supplementary material for: A human progeria-associated BAF-1 mutation modulates gene expression and accelerates aging in C. elegans
Source: EMBO J. 2024 Oct 4;43(22):18. doi: 10.1038/s44318-024-00261-8 (PMC11574047; doi:10.1038/s44318-024-00261-8)
Supplement: Supplementary file 5 — Table EV5 [file 44318_2024_261_MOESM5_ESM.pdf]

Table EV5

Sample	Dam fusion	*baf-1*	Tissue	Experiment	Raw.reads	Cut.reads
BN1047R1	Dam::BAF-1	WT	Hyp	BAF-1 DamID	18740263	3005144
BN1047R2	Dam::BAF-1	WT	Hyp	BAF-1 DamID	23215175	9272778
BN1047R3	Dam::BAF-1	WT	Hyp	BAF-1 DamID	27642934	9395426
BN561R1	GFP::Dam	WT	Hyp	BAF-1 DamID	20511930	3382859
BN561R2	GFP::Dam	WT	Hyp	BAF-1 DamID	11999338	3534312
BN561R3	GFP::Dam	WT	Hyp	BAF-1 DamID	6835704	2211321
BN1052R1	Dam::BAF-1	WT	Int	BAF-1 DamID	39808838	7620330
BN1052R2	Dam::BAF-1	WT	Int	BAF-1 DamID	58252001	9750214
BN1052R3	Dam::BAF-1	WT	Int	BAF-1 DamID	40395718	6985997
BN1051R1	GFP::Dam	WT	Int	BAF-1 DamID	11620807	1957742
BN1051R2	GFP::Dam	WT	Int	BAF-1 DamID	66542014	12815500
BN1051R3	GFP::Dam	WT	Int	BAF-1 DamID	39093117	5577439
BN1050R1	Dam::BAF-1(G12T)	*baf-1(G12T)*	Hyp	BAF-1 DamID	13500562	3095446
BN1050R2	Dam::BAF-1(G12T)	*baf-1(G12T)*	Hyp	BAF-1 DamID	28347252	8574994
BN1050R3	Dam::BAF-1(G12T)	*baf-1(G12T)*	Hyp	BAF-1 DamID	10603157	3537523
BN1048R1	GFP::Dam	*baf-1(G12T)*	Hyp	BAF-1 DamID	14039486	2459948
BN1048R2	GFP::Dam	*baf-1(G12T)*	Hyp	BAF-1 DamID	19416940	4604504
BN1048R3	GFP::Dam	*baf-1(G12T)*	Hyp	BAF-1 DamID	13719184	4591705
BN1054R1	Dam::BAF-1(G12T)	*baf-1(G12T)*	Int	BAF-1 DamID	45560629	6473550
BN1054R2	Dam::BAF-1(G12T)	*baf-1(G12T)*	Int	BAF-1 DamID	55998615	7228206
BN1054R3	Dam::BAF-1(G12T)	*baf-1(G12T)*	Int	BAF-1 DamID	10156315	3489919
BN1053R1	GFP::Dam	*baf-1(G12T)*	Int	BAF-1 DamID	10397627	2119217
BN1053R2	GFP::Dam	*baf-1(G12T)*	Int	BAF-1 DamID	6735160	1825474
BN1053R3	GFP::Dam	*baf-1(G12T)*	Int	BAF-1 DamID	45604517	5500716
BN1414R1	Dam::RPB-6	WT	Hyp	RAPID	55151578	49896285
BN1414R2	Dam::RPB-6	WT	Hyp	RAPID	29472050	27283523
BN561R1	GFP::Dam	WT	Hyp	RAPID	25851173	23996691
BN561R2	GFP::Dam	WT	Hyp	RAPID	34142422	31912638
BN1415R1	Dam::RPB-6	WT	Int	RAPID	38725533	36232658
BN1415R2	Dam::RPB-6	WT	Int	RAPID	51270952	46191739
BN1051R1	GFP::Dam	WT	Int	RAPID	27495129	25830819
BN1051R2	GFP::Dam	WT	Int	RAPID	24825994	23123070
BN1416R1	Dam::RPB-6	*baf-1(G12T)*	Hyp	RAPID	76541282	68788560
BN1416R2	Dam::RPB-6	*baf-1(G12T)*	Hyp	RAPID	107439981	100096170
BN1048R1	GFP::Dam	*baf-1(G12T)*	Hyp	RAPID	22861824	21200127
BN1048R2	GFP::Dam	*baf-1(G12T)*	Hyp	RAPID	28267352	26364680
BN1417R1	Dam::RPB-6	*baf-1(G12T)*	Int	RAPID	32389256	30146272
BN1417R2	Dam::RPB-6	*baf-1(G12T)*	Int	RAPID	33315508	30389405
BN1053R1	GFP::Dam	*baf-1(G12T)*	Int	RAPID	30530830	28777590
BN1053R2	GFP::Dam	*baf-1(G12T)*	Int	RAPID	39309242	36788497

Individual DamID samples obtained in this study

Mapped.reads	GATC.reads	Percentage
1485068	1481972	7.9
2350374	2322595	10.0
2982162	2933349	10.6
1982157	1979881	9.7
1847526	1838635	15.3
1473010	1466550	21.5
896733	890652	2.2
536843	515492	0.9
4653416	4609737	11.4
1466782	1465633	12.6
8997154	8973703	13.5
4257133	4242468	10.9
1517815	1513434	11.2
1713384	1629751	5.7
1730454	1719304	16.2
1836312	1833763	13.1
2860379	2847206	14.7
3150279	3143647	22.9
2889857	2866010	6.3
3259482	2998322	5.4
2179779	2159861	21.3
1533011	1531288	14.7
1154150	1150780	17.1
3849699	3842500	8.4
7327585	7259801	13.2
11135772	11125581	37.7
15318783	15312243	59.2
19855436	19849763	58.1
10573724	10567462	27.3
15459449	15420825	30.1
15911235	15907256	57.9
13559944	13556832	54.6
3552440	3430318	4.5
27493236	27451137	25.6
12544566	12538120	54.8
16859072	16853186	59.6
10307381	10294746	31.8
10086027	10016998	30.1
17602729	17598709	57.6
21988543	21983661	55.9
