# Supplementary material for: A human progeria-associated BAF-1 mutation modulates gene expression and accelerates aging in C. elegans
Source: EMBO J. 2024 Oct 4;43(22):18. doi: 10.1038/s44318-024-00261-8 (PMC11574047; doi:10.1038/s44318-024-00261-8)

Appendix

A human progeria-associated BAF-1 mutation modulates gene expression and accelerates aging in *C. elegans*

Raquel Romero-Bueno, Adrián Fragoso-Luna, Cristina Ayuso, Nina Mellmann, Alan Kavsek, Christian G. Riedel, Jordan D. Ward and Peter Askjaer

Table of Content

Appendix Figure Legends	Page 2
Appendix Figure S1	Page 3
Appendix Figure S2	Page 4
Appendix Figure S3	Page 5
Appendix Figure S4	Page 6
Appendix Figure S5	Page 7

Appendix Figures Legends

Appendix Figure S1. BAF-1 and BAF-1(G12T) but not LMN-1 nor EMR-1 are present in mature sperm. Confocal micrographs of males (A, E) and hermaphrodites (B-D, F) expressing fluorescent proteins as indicated. Animals were wild-type for *baf-1*, except *baf-1(G12T)* in C and F. Spermathecae of hermaphrodites and gonads of males are outlined; arrows indicate examples of sperm.

Appendix Figure S2. Nuclear morphology deteriorates faster in *baf-1(G12T)* mutants. (A-D) Morphology of hypodermal nuclei was classified on day 8 of adulthood in strains expressing GFP::LMN-1 (green) and mCh::HIS-58 (magenta) (A, B) or EMR-1:.mCh (C, D). Confocal micrographs (A, C) and combined data from 4 independent experiments (B, D) are shown. Scale bars represent 10 µm (A) or 5 µm (C). Classes I-IV are defined in Materials and Methods; briefly, class I corresponds to nuclei with smooth, regular NE morphology and classes II-IV represent increasingly irregular morphologies. The number of nuclei analyzed, and statistical tests are reported in Table EV1. (E-F) Quantitative RT-PCR was performed on whole-animal RNA from WT and *baf-1(G12T)* animals. (E) Average Cq values for the control genes *tba-1*, *pmp-3* and Y45F10D.4 (Zhang et al. 2012). (F) Expression of *baf-1/baf-1(G12T)*, *lmn-1* and *emr-1* relative to *tba-1*. Error bars in (E-F) represent standard deviation.

Appendix Figure S3. *baf-1(G12T)* worms are hypersensitive to GFP tagging of LMN-1. (A) Embryonic lethality was quantified for hermaphrodites carrying combinations of *baf-1(G12T)* and *gfp::lmn-1(jf98)* as indicated. Each symbol represents the average per 3-5 worms. (B) Selected time points from confocal timelapse recording of 1 control and 3 *baf-1(G12T)* embryos expressing endogenously tagged GFP::LMN-1 (green; allele *yc32*) and mCh::HIS-58 (magenta). Arrows indicate failures in chromosome segregation. Scale bars represent 10 µm.

Appendix Figure S4. Chromatin accessibility is unaffected in *baf-1(G12T)* mutants. (A) Spearman correlation values for individual GFP::Dam samples with reads binned in 2 kb regions. Note that the 2 tissues (hypodermis = hyp; intestine = int) cluster separately and, within each tissue, the correlations between wild-type and *baf-1(G12T)* are higher than the correlations between the three replicas. (B) Genome browser views of normalized GFP::Dam reads per 2 kb bins for chrII.

Appendix Figure S5. Variation in BAF-1 chromatin association is higher between tissues than between wild-type BAF-1 and mutant BAF-1(G12T). (A) Pearson correlation values for log2 values (Dam::BAF-1/GFP::Dam or Dam::BAF-1(G12T)/GFP::Dam) binned in 100 kb regions. Note that the 2 tissues (hypodermis = hyp; intestine = int) cluster separately and, within each tissue, the correlations between wild-type and *baf-1(G12T)* are higher than the correlations between the three replicas. (B, C) Normalized log2 values of Dam::BAF-1/GFP::Dam and Dam::BAF-1(G12T)/GFP::Dam ratios per 100 kb bins for the entire genome (B) and per 2 kb bins as genome browser view of 500 kb region from left arm of chrI (C). For clarity, the curves in B represent rolling means across 3 consecutive bins. Grey bars above each track in C indicate enriched association to BAF-1 or BAF-1(G12T) (FDR < 0.05). Examples of regions enriched for association to either BAF-1 or BAF-1(G12T) in the intestine are outlines with dashed boxed.

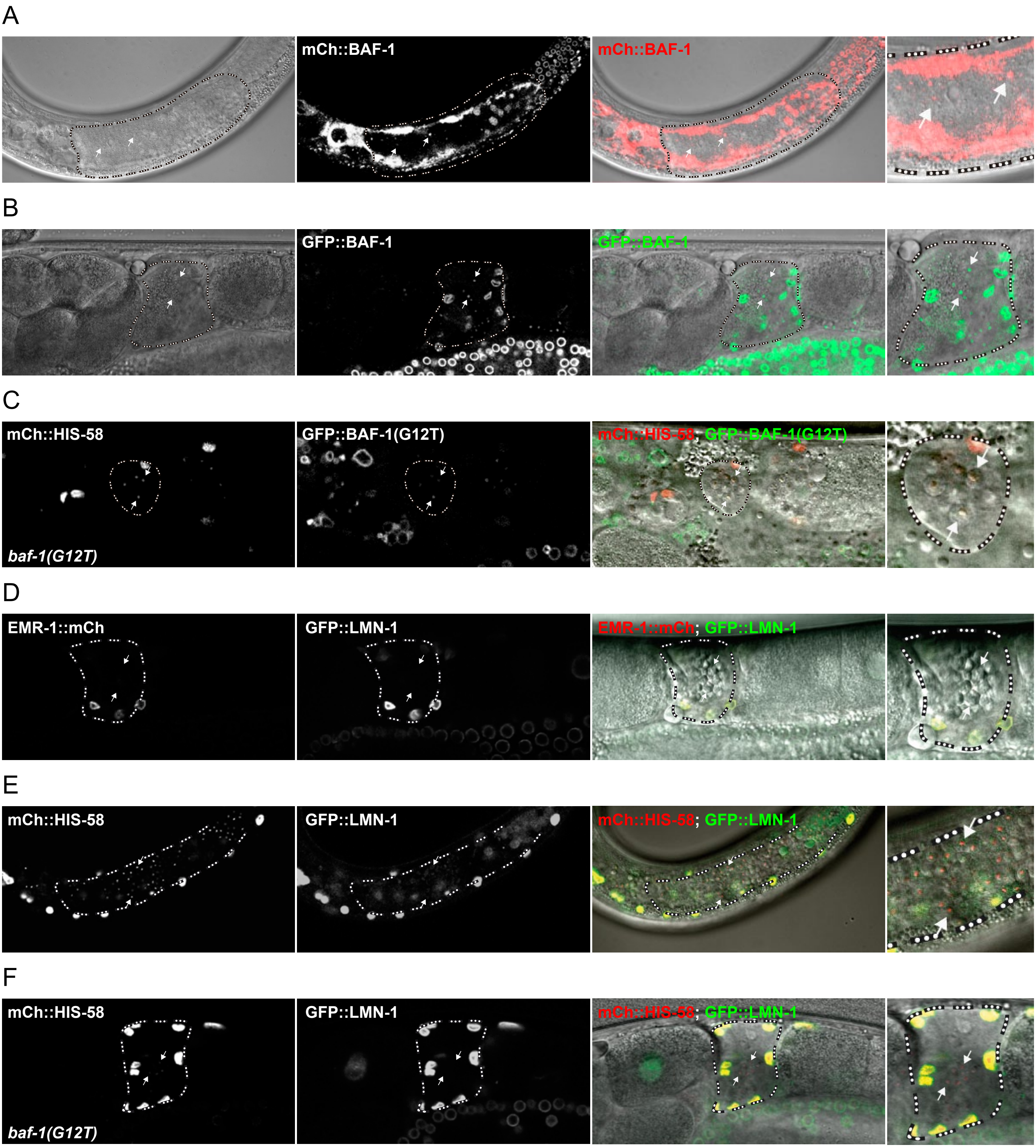
A
mCh::BAF-1
mCh::BAF-1
B
GFP::BAF-1
GFP::BAF-1
C
mCh::HIS-58
baf-1(G12T)
GFP::BAF-1(G12T)
mCh::HIS-58; GFP::BAF-1(G12T)
D
EMR-1::mCh
GFP::LMN-1
EMR-1::mCh; GFP::LMN-1
E
mCh::HIS-58
GFP::LMN-1
mCh::HIS-58; GFP::LMN-1
F
mCh::HIS-58
baf-1(G12T)
GFP::LMN-1
mCh::HIS-58; GFP::LMN-1

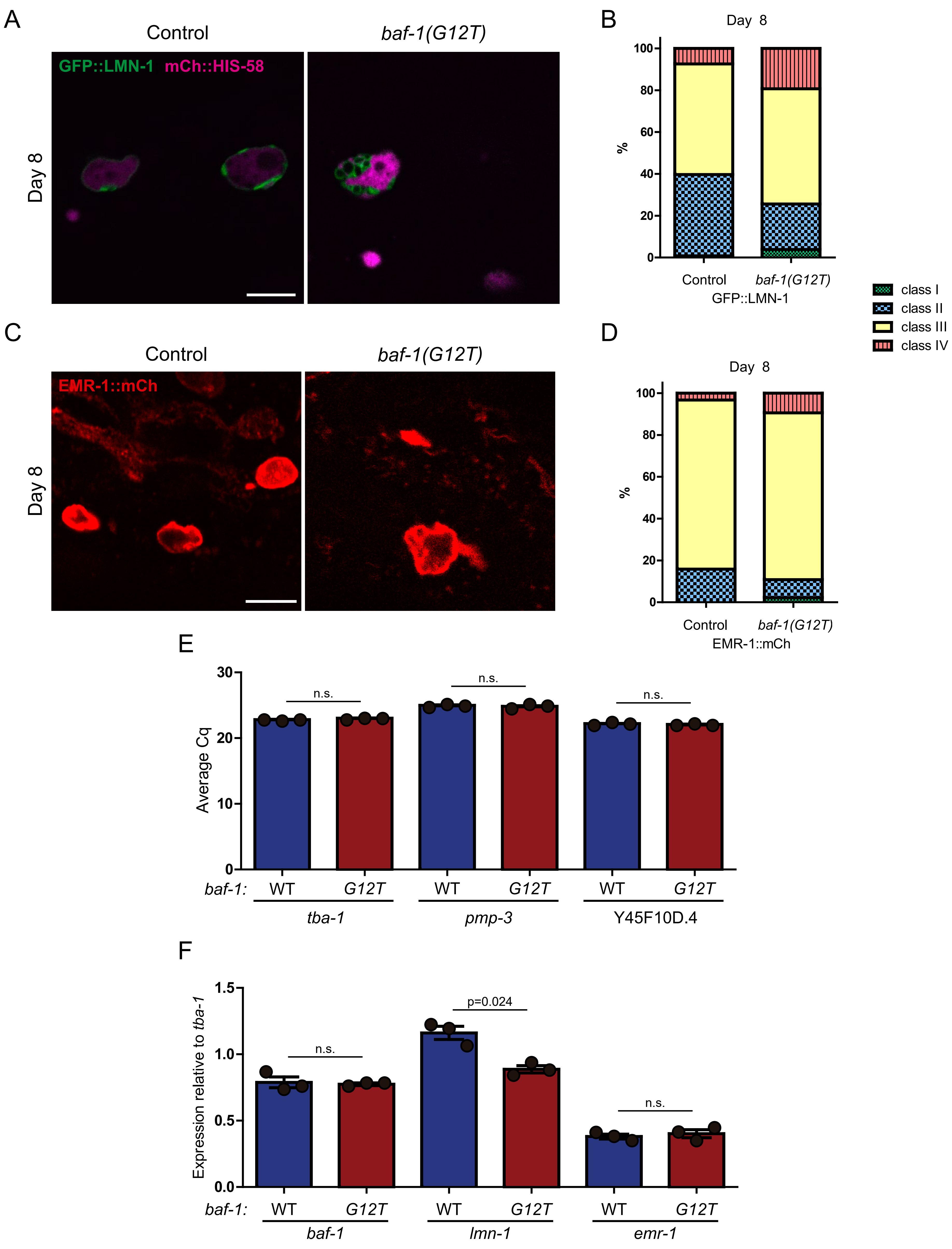
A
Control
baf-1(G12T)
GFP::LMN-1 mCh::HIS-58
Day 8
B
Day 8
%
100
80
60
40
20
0
Control
baf-1(G12T)
GFP::LMN-1
class I
class II
class III
class IV
C
Control
baf-1(G12T)
EMR-1::mCh
Day 8
D
Day 8
%
Control
baf-1(G12T)
EMR-1::mCh
E
Average Cq
30
20
10
0
n.s.
baf-1:
WT
G12T
tba-1
pmp-3
Y45F10D.4
F
Expression relative to tba-1
1.5
1.0
0.5
0.0
p=0.024
baf-1
lmn-1
emr-1

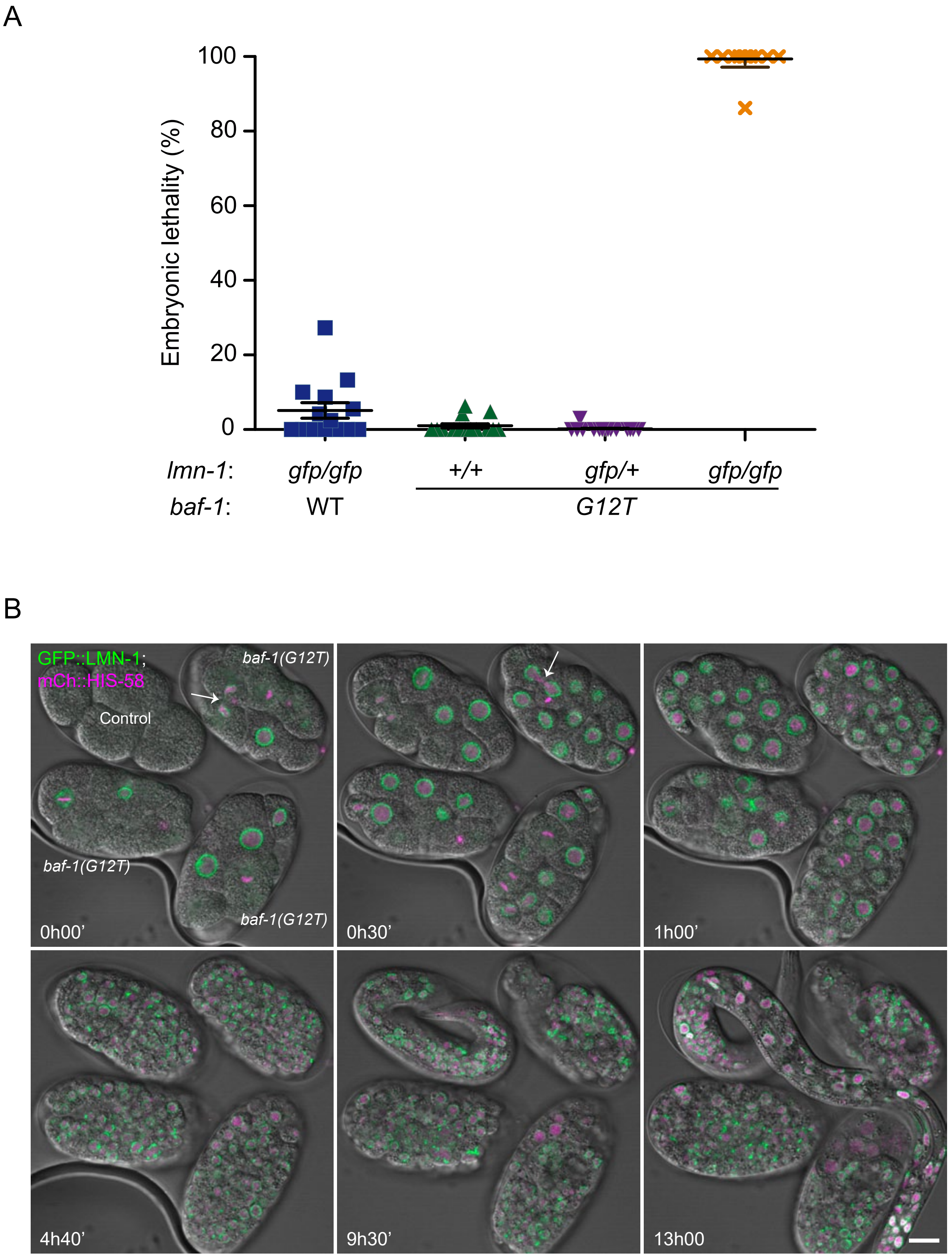
A
Embryonic lethality (%)
100
80
60
40
20
0
lmn-1:
gfp/gfp
+/+
gfp/+
gfp/gfp
baf-1:
WT
G12T
B
GFP::LMN-1;
mCh::HIS-58
Control
baf-1(G12T)
baf-1(G12T)
baf-1(G12T)
0h00’
0h30’
1h00’
4h40’
9h30’
13h00

A

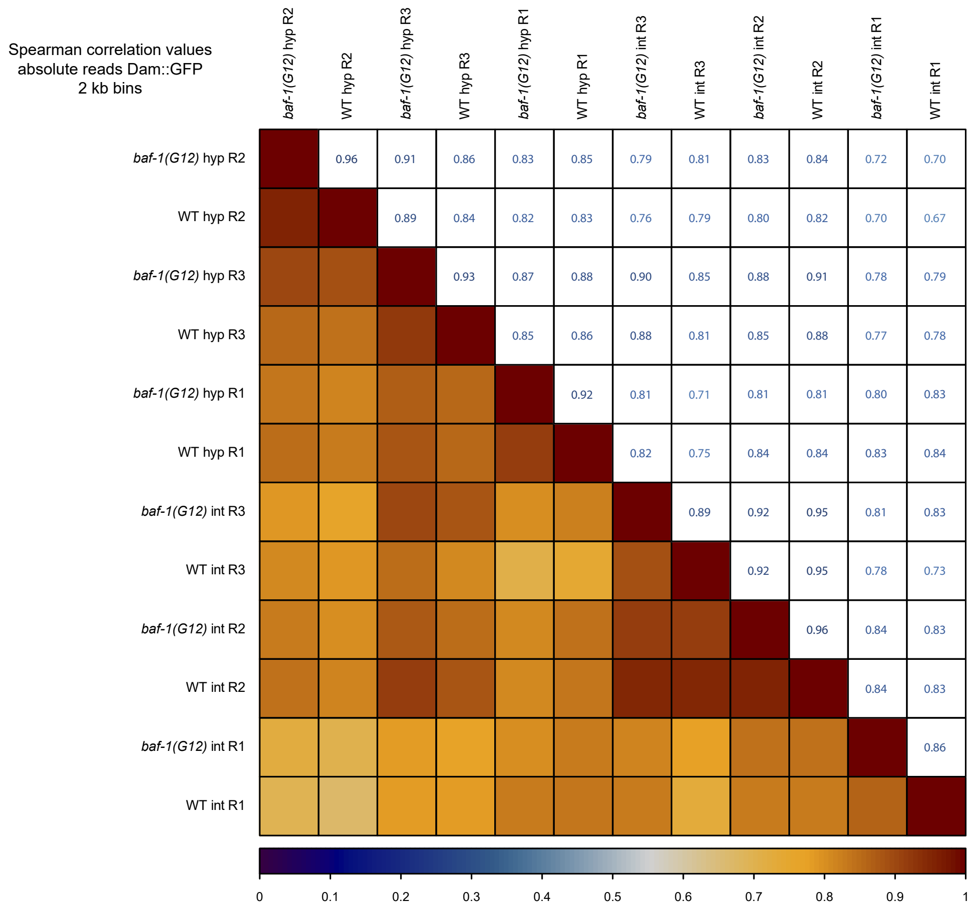
Spearman correlation values
absolute reads Dam::GFP
2 kb bins
baf-1(G12) hyp R2
WT hyp R2
baf-1(G12) hyp R3
WT hyp R3
baf-1(G12) hyp R1
WT hyp R1
baf-1(G12) int R3
WT int R3
baf-1(G12) int R2
WT int R2
baf-1(G12) int R1
WT int R1
0.96 0.91 0.86 0.83 0.85 0.79 0.81 0.83 0.84 0.72 0.70
0.89 0.84 0.82 0.83 0.76 0.79 0.80 0.82 0.70 0.67
0.93 0.87 0.88 0.90 0.85 0.88 0.91 0.78 0.79
0.85 0.86 0.88 0.81 0.85 0.88 0.77 0.78
0.92 0.81 0.71 0.81 0.81 0.80 0.83
0.82 0.75 0.84 0.84 0.83 0.84
0.89 0.92 0.95 0.81 0.83
0.92 0.95 0.78 0.73
0.96 0.84 0.83
0.84 0.83
0.86
0 0.1 0.2 0.3 0.4 0.5 0.6 0.7 0.8 0.9 1

B

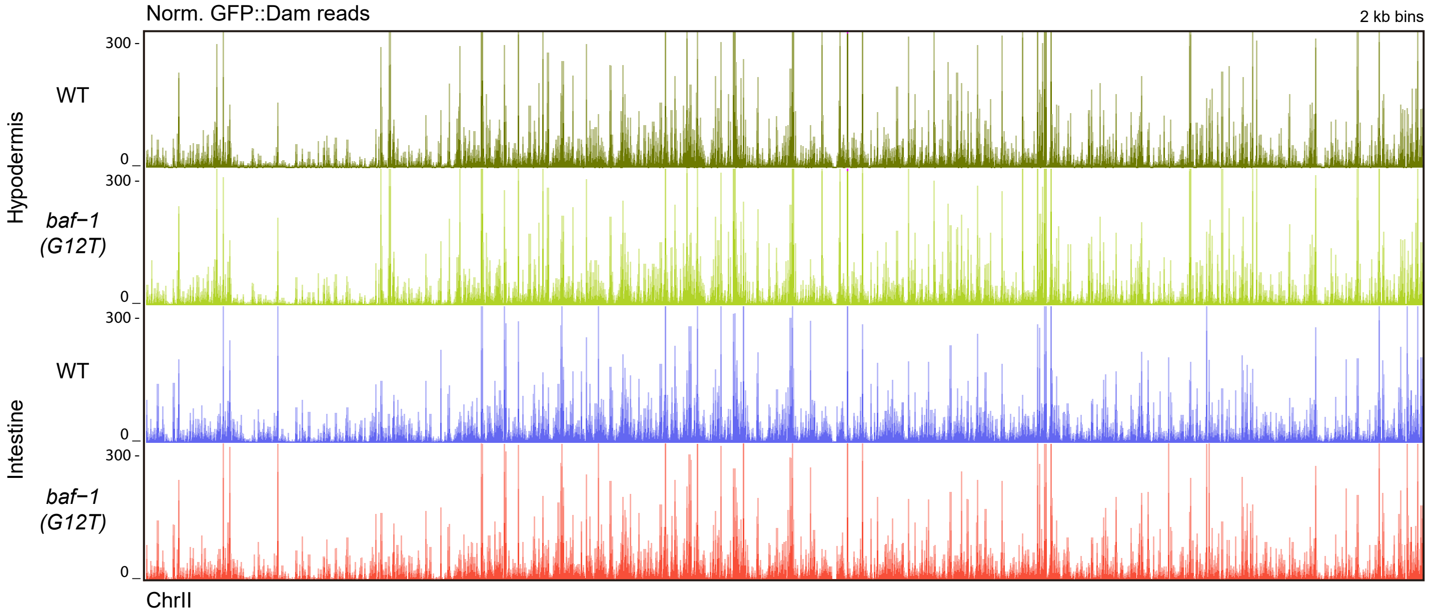
Norm. GFP::Dam reads
2 kb bins
Hypodermis
WT
baf−1 (G12T)
Intestine
WT
baf−1 (G12T)
300 -
0
ChrII

Appendix Figure S5

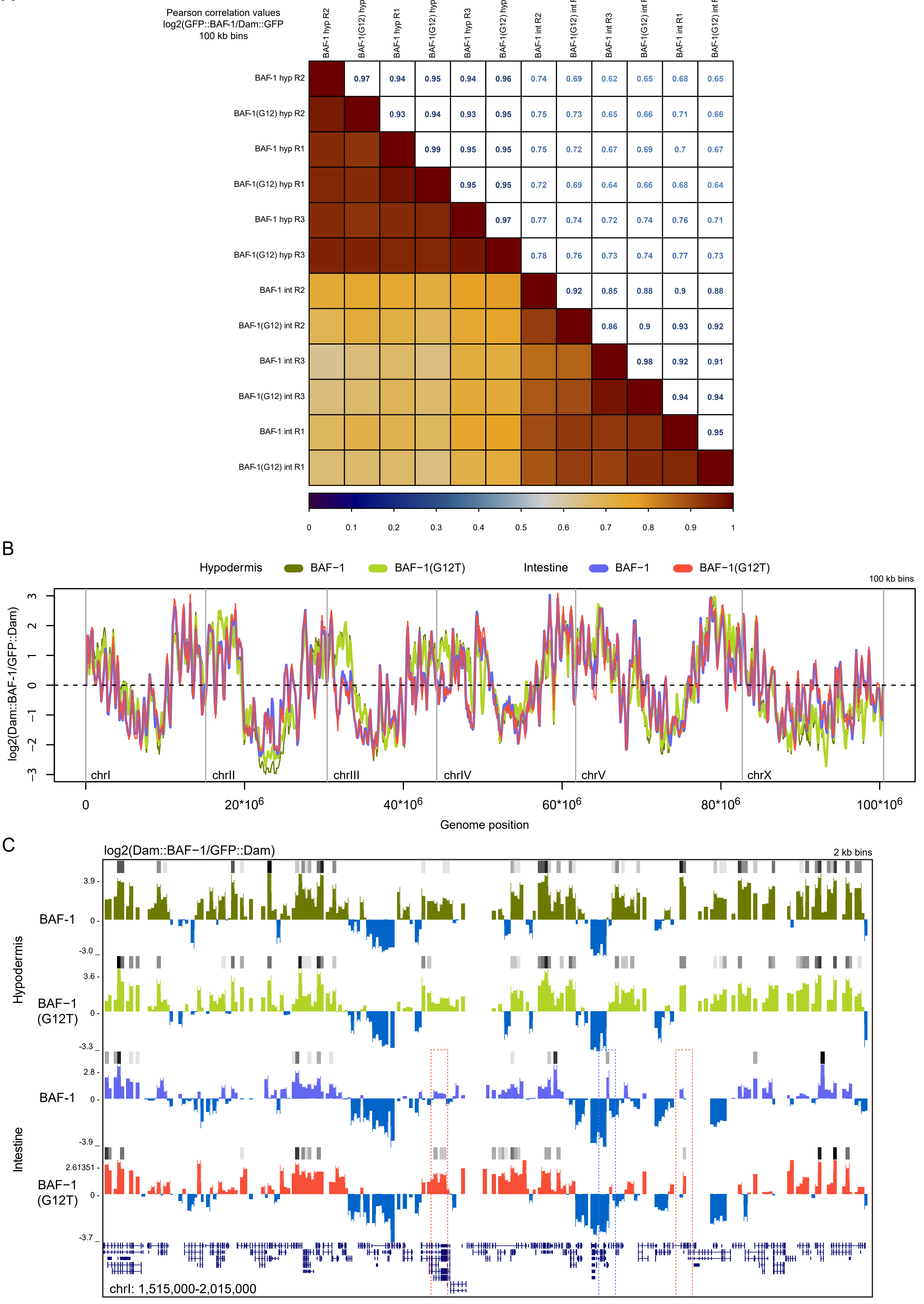

Supplement: Supplementary file 6 — Appendix [file 44318_2024_261_MOESM6_ESM.pdf]
