# Supplementary material for: A human progeria-associated BAF-1 mutation modulates gene expression and accelerates aging in C. elegans
Source: EMBO J. 2024 Oct 4;43(22):18. doi: 10.1038/s44318-024-00261-8 (PMC11574047; doi:10.1038/s44318-024-00261-8)
Supplement: Supplementary file 10 — Movie EV1 [file 44318_2024_261_MOESM10_ESM.zip › Movie_EV1.docx]

**Movie EV1. Abnormal localization of GFP::LMN-1 and chromosome segregation defects caused by *baf-1(G12T)*.** Control (left) and *baf-1(G12T)* (right) embryos expressing GFP::LMN-1 (green) and mCh::HIS-58 (magenta) were observed by spinning disk confocal microscopy every 12.5 sec. Each time point represents maximum projections of 17 confocal slices. Corresponds to Figure 3B.
