# Supplementary material for: A human progeria-associated BAF-1 mutation modulates gene expression and accelerates aging in C. elegans
Source: EMBO J. 2024 Oct 4;43(22):18. doi: 10.1038/s44318-024-00261-8 (PMC11574047; doi:10.1038/s44318-024-00261-8)
Supplement: Supplementary file 11 — Movie EV2 [file 44318_2024_261_MOESM11_ESM.zip › Movie_EV2.docx]

**Movie EV2. Abnormal localization of GFP::BAF-1(G12T) and chromosome segregation defects caused by *baf-1(G12T)*.** Control GFP::BAF-1 (left) and GFP::BAF-1(G12T) (right) embryos co-expressing mCh::HIS-58 (magenta; GFP in green) were observed by spinning disk confocal microscopy every 20.4 sec. Each time point represents maximum projections of 17 confocal slices. Corresponds to Figure 3D.
