# Supplementary figures and images for: A human progeria-associated BAF-1 mutation modulates gene expression and accelerates aging in C. elegans
Source: EMBO J. 2024 Oct 4;43(22):18. doi: 10.1038/s44318-024-00261-8 (PMC11574047; doi:10.1038/s44318-024-00261-8)

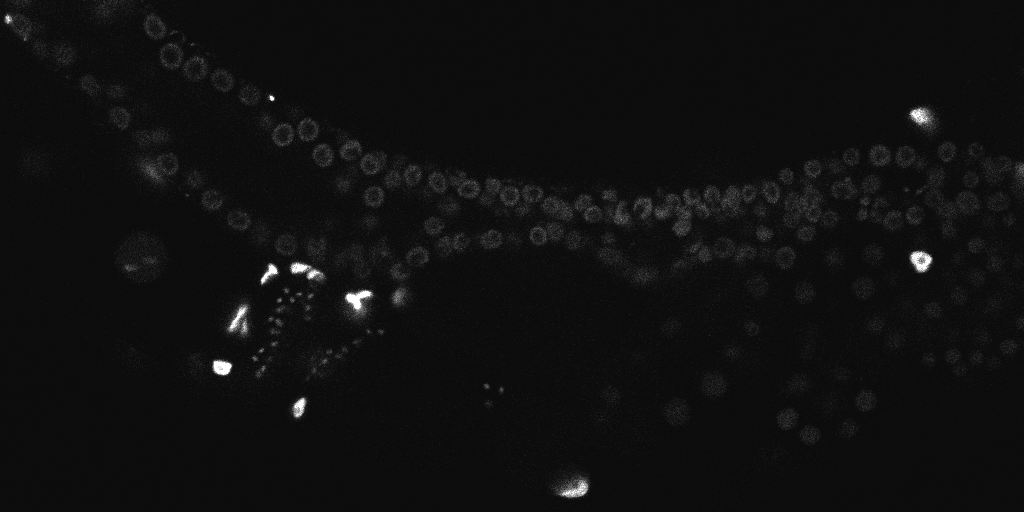

Supplement: Supplementary file 12 — Source data Fig. 1 [file 44318_2024_261_MOESM12_ESM.zip › Figure 1/1C/BN189_MUT_day1_8bit.tif]

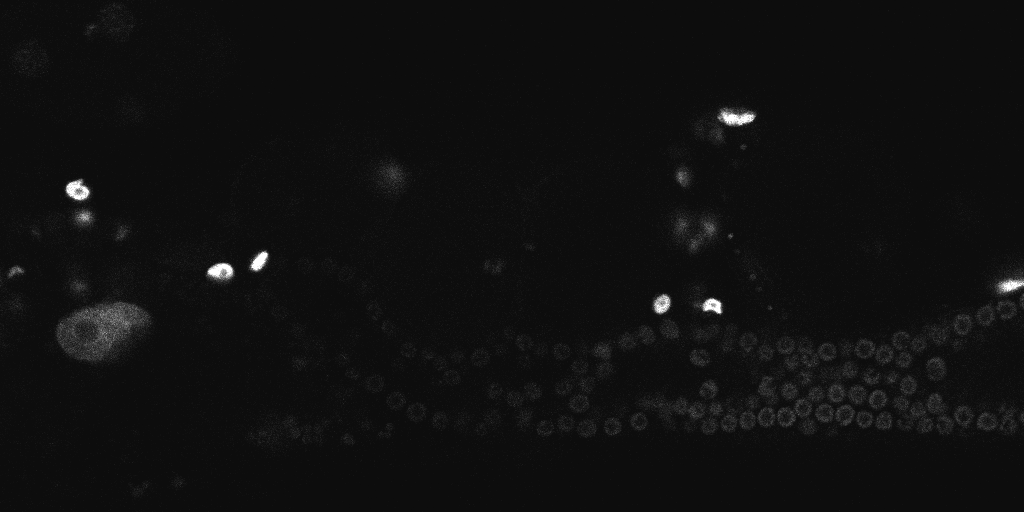

Supplement: Supplementary file 12 — Source data Fig. 1 [file 44318_2024_261_MOESM12_ESM.zip › Figure 1/1C/BN189_MUT_day2_8bit.tif]

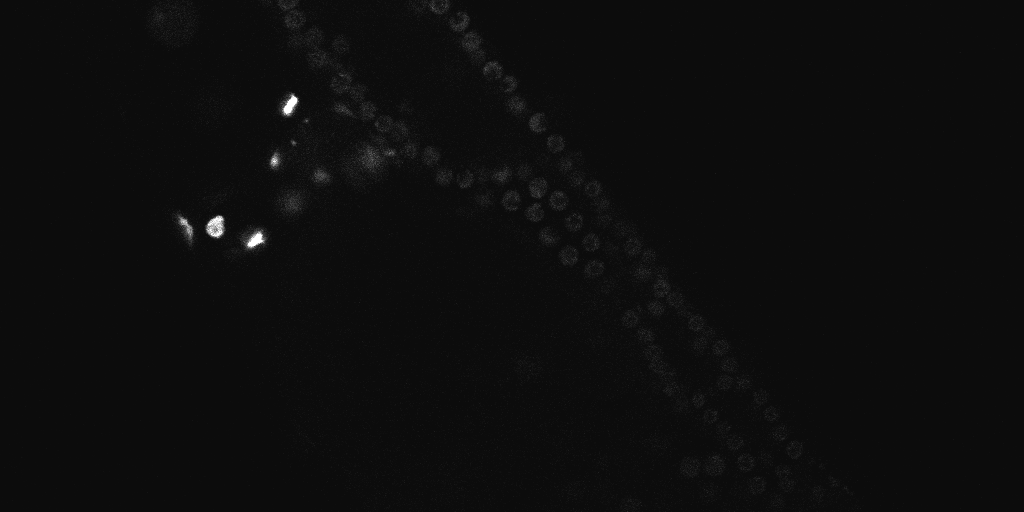

Supplement: Supplementary file 12 — Source data Fig. 1 [file 44318_2024_261_MOESM12_ESM.zip › Figure 1/1C/BN189_MUT_day3_8bit.tif]

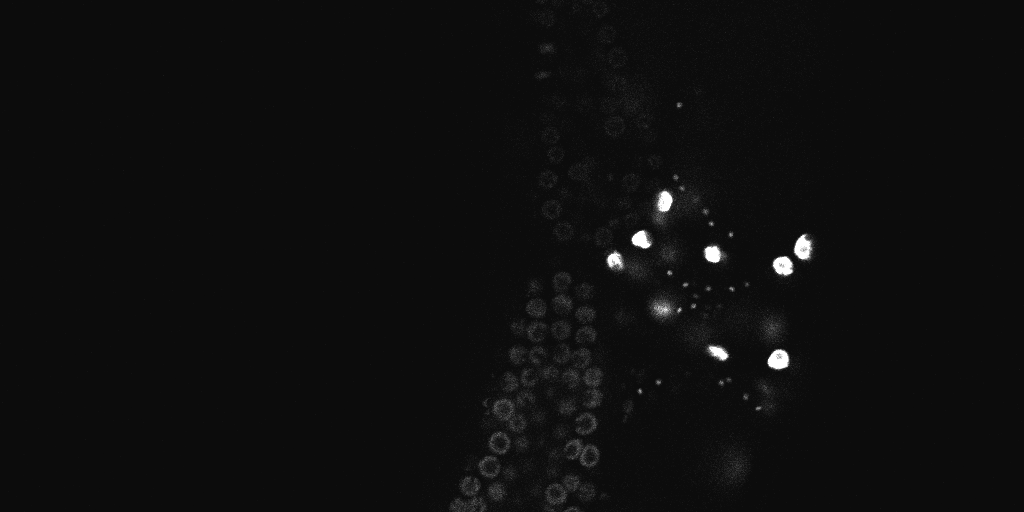

Supplement: Supplementary file 12 — Source data Fig. 1 [file 44318_2024_261_MOESM12_ESM.zip › Figure 1/1C/BN874_MUT_day1_8bit.tif]

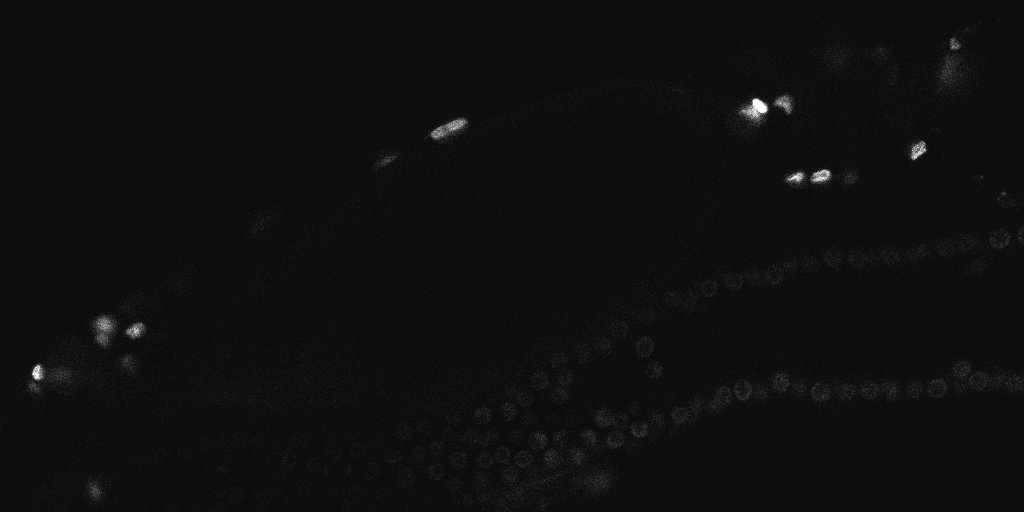

Supplement: Supplementary file 12 — Source data Fig. 1 [file 44318_2024_261_MOESM12_ESM.zip › Figure 1/1C/BN874_MUT_day2_8bit.tif]

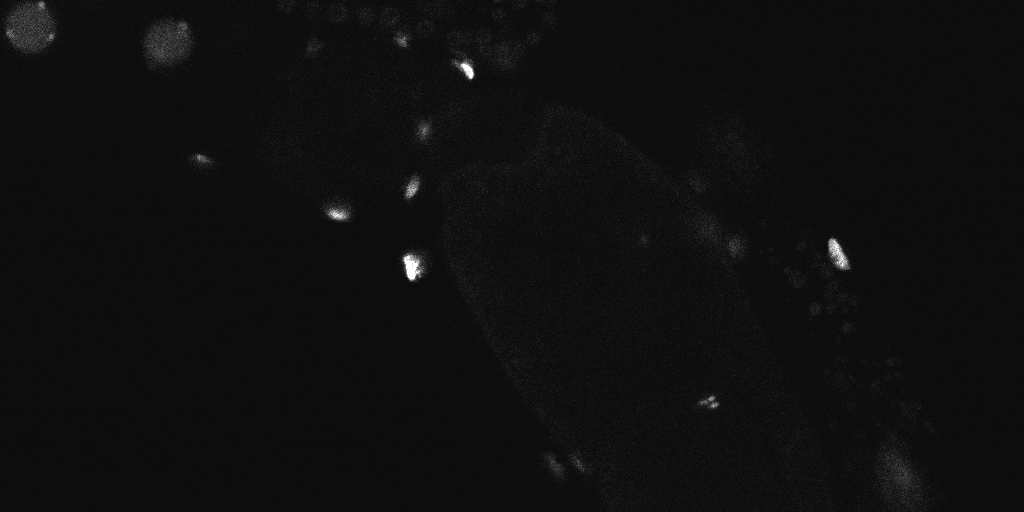

Supplement: Supplementary file 12 — Source data Fig. 1 [file 44318_2024_261_MOESM12_ESM.zip › Figure 1/1C/BN874_MUT_day3_8bit.tif]

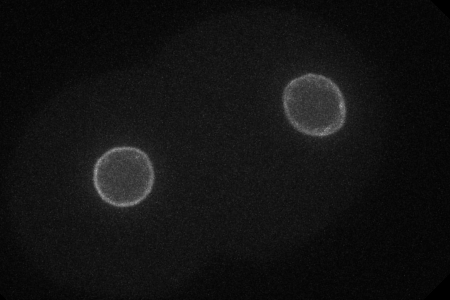

Supplement: Supplementary file 14 — Source data Fig. 3 [file 44318_2024_261_MOESM14_ESM.zip › Figure 3/3B/BN869_WT_GFP_LMN-1.tif]

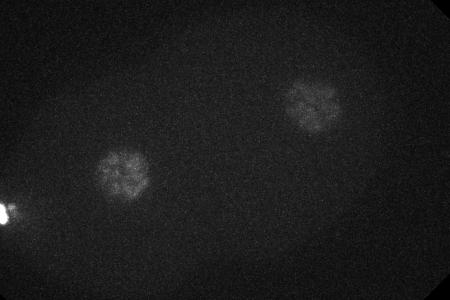

Supplement: Supplementary file 14 — Source data Fig. 3 [file 44318_2024_261_MOESM14_ESM.zip › Figure 3/3B/BN869_WT_mCh_HIS-58.tif]

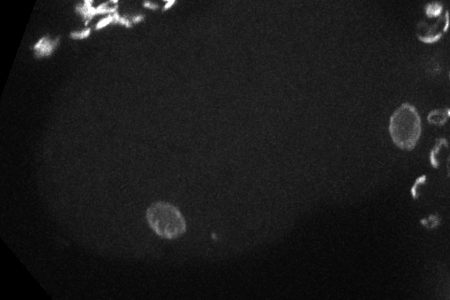

Supplement: Supplementary file 14 — Source data Fig. 3 [file 44318_2024_261_MOESM14_ESM.zip › Figure 3/3B/BN870_MUT_GFP_LMN-1.tif]

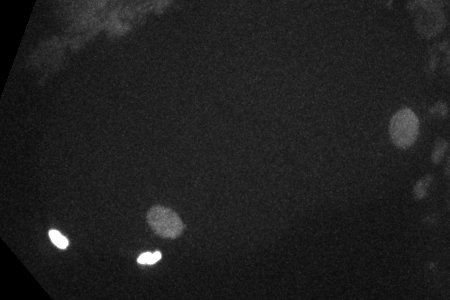

Supplement: Supplementary file 14 — Source data Fig. 3 [file 44318_2024_261_MOESM14_ESM.zip › Figure 3/3B/BN870_MUT_mCh_HIS-58.tif]

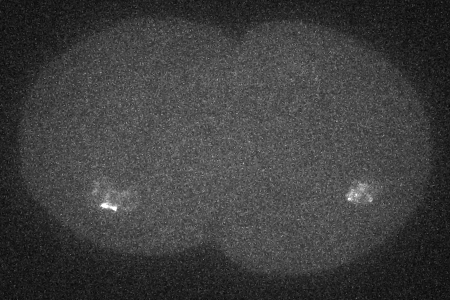

Supplement: Supplementary file 14 — Source data Fig. 3 [file 44318_2024_261_MOESM14_ESM.zip › Figure 3/3D/BN1188_MUT_GFP_BAF-1_G12T.tif]

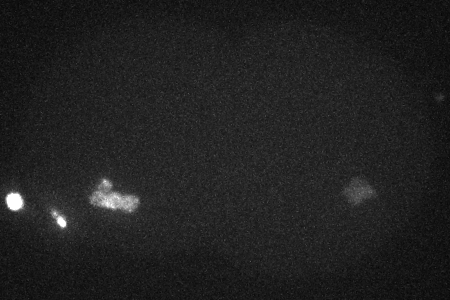

Supplement: Supplementary file 14 — Source data Fig. 3 [file 44318_2024_261_MOESM14_ESM.zip › Figure 3/3D/BN1188_MUT_mCh_HIS-58.tif]

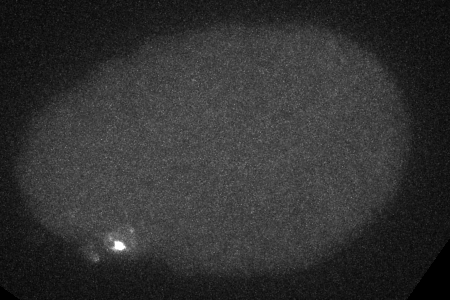

Supplement: Supplementary file 14 — Source data Fig. 3 [file 44318_2024_261_MOESM14_ESM.zip › Figure 3/3D/BN599_WT_GFP_BAF-1.tif]

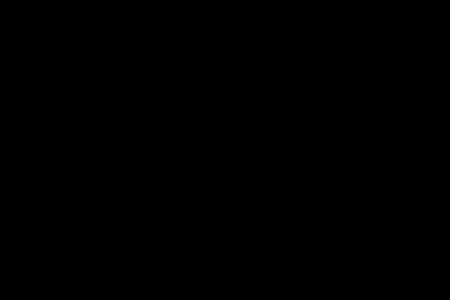

Supplement: Supplementary file 14 — Source data Fig. 3 [file 44318_2024_261_MOESM14_ESM.zip › Figure 3/3D/BN599_WT_mCh_HIS-58.tif]
