# Supplementary material for: A human progeria-associated BAF-1 mutation modulates gene expression and accelerates aging in C. elegans
Source: EMBO J. 2024 Oct 4;43(22):18. doi: 10.1038/s44318-024-00261-8 (PMC11574047; doi:10.1038/s44318-024-00261-8)
Supplement: Supplementary file 16 — Expanded View Figures [file 44318_2024_261_MOESM16_ESM.pdf]

Expanded View Figures

Figure EV1. *baf-1(G12T)* mutants have normal viability and lifespan at 20 °C.

(**A**) The predicted 3-dimensional structure of *C. elegans* BAF-1 (rainbow-colored; Uniprot Q03565) was superimposed onto the structure of human BAF (magenta; Uniprot O75531) using iCn3D Structure Viewer (Wang et al, 2022) (https://www.ncbi.nlm.nih.gov/Structure/icn3d/). Residue 12 is highlighted in green. (**B**, **C**) The egg laying rate (**B**) and embryonic viability (**C**) were determined for hermaphrodites grown at 20 °C, 25 °C or shifted from 20 °C to 25 °C when reaching adulthood. (**D**) Lifespan assay of wild-type and *baf-1(G12T)* hermaphrodites at 20 °C. The number of animals analyzed is indicated in brackets. *P* values from two-sided t-test (**B**, **C**) and Chi-square test (**D**) are indicated; *p* values ≥ 0.05 are considered not significant (n.s.).

© The Author(s)

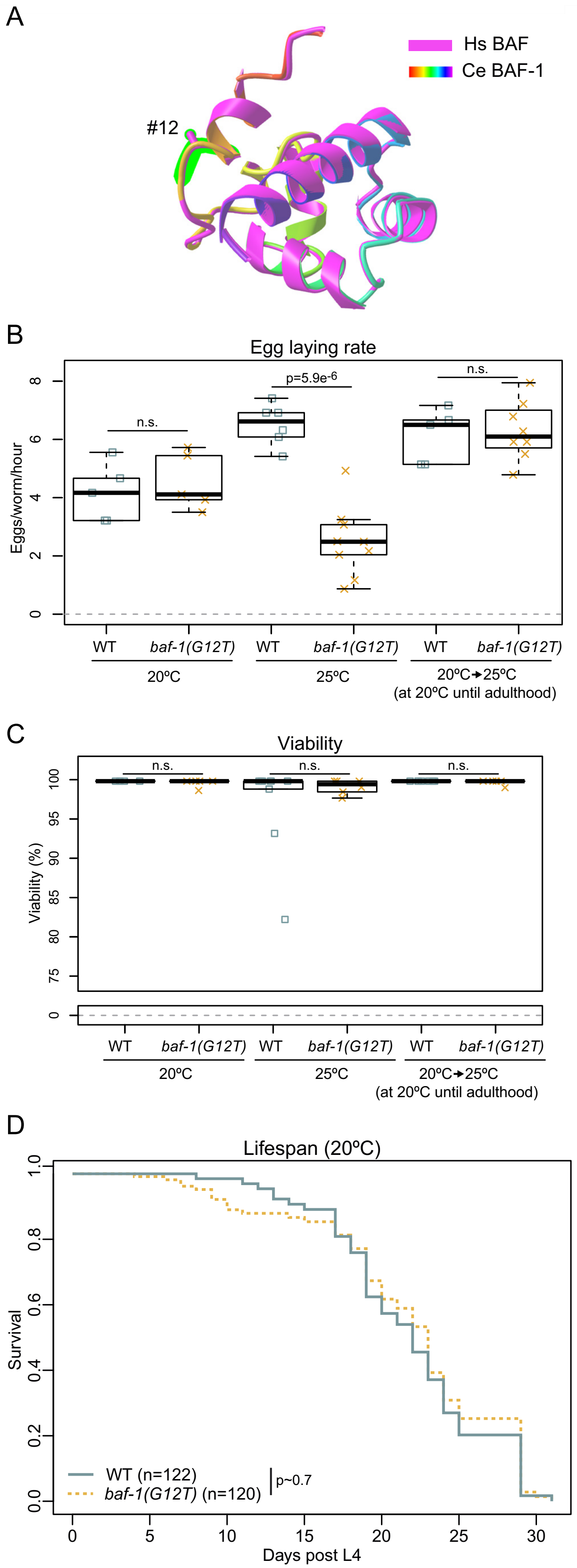
A
Hs BAF
Ce BAF-1
#12
B
Egg laying rate
Eggs/worm/hour
0
2
4
6
8
n.s.
p=5.9e-6
n.s.
WT
baf-1(G12T)
WT
baf-1(G12T)
WT
baf-1(G12T)
20ºC
25ºC
20ºC→25ºC
(at 20ºC until adulthood)
C
Viability
Viability (%)
0
75
80
85
90
95
100
n.s.
n.s.
n.s.
WT
baf-1(G12T)
WT
baf-1(G12T)
WT
baf-1(G12T)
20ºC
25ºC
20ºC→25ºC
(at 20ºC until adulthood)
D
Lifespan (20ºC)
Survival
0.0
0.2
0.4
0.6
0.8
1.0
WT (n=122)
baf-1(G12T) (n=120)
p~0.7
0
5
10
15
20
25
30
Days post L4

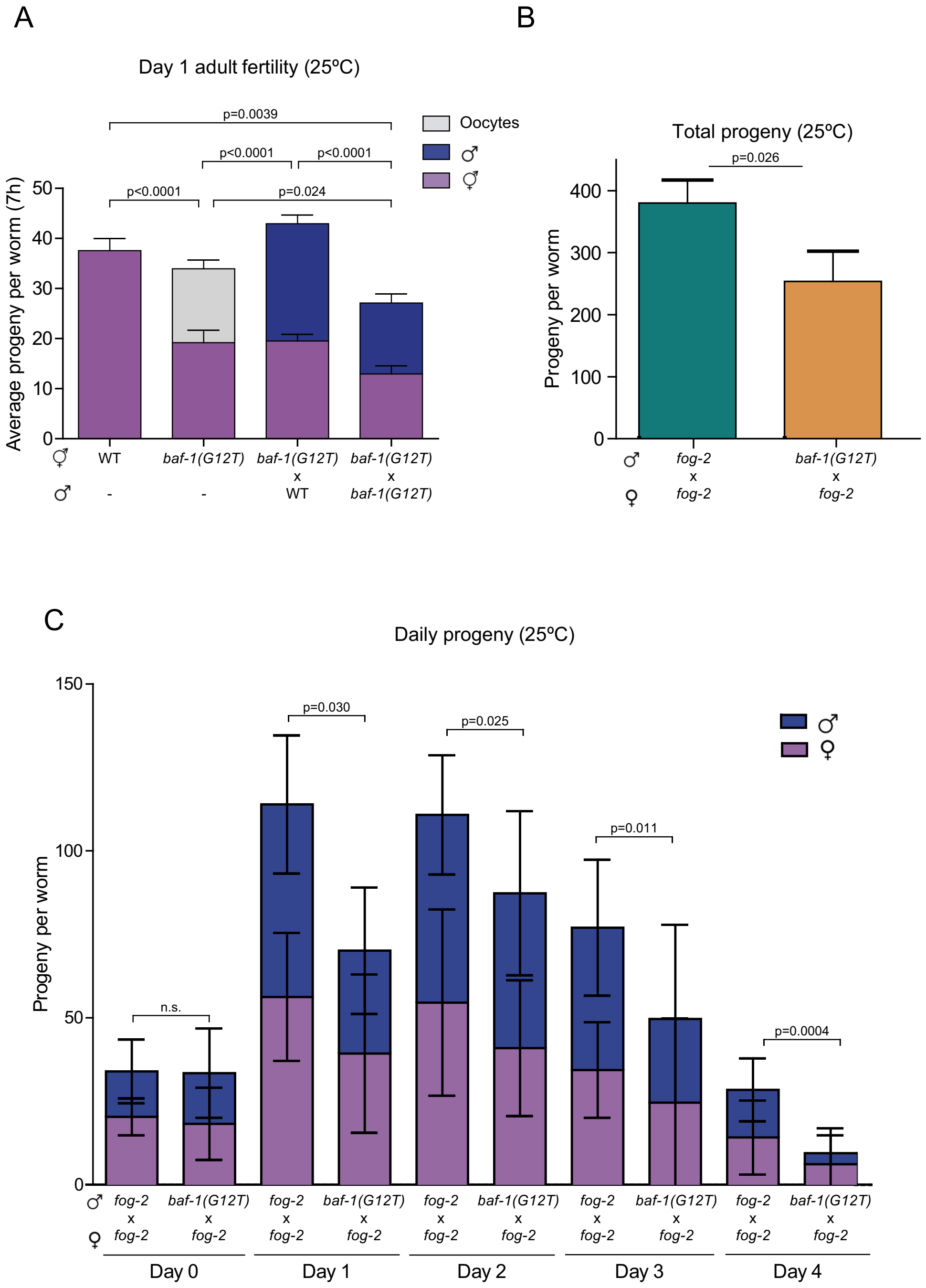

Figure EV2. Sperm defect caused by *baf-1(G12T)* mutation.

(**A**) Progeny produced at 25 °C by day 1 wild-type and *baf-1(G12T)* adults either unmated or mated with wild-type or *baf-1(G12T)* males as indicated. (**B**, **C**) Total (**B**) and daily (**C**) brood size produced at 25 °C by feminized *fog-2* animals mated with either *fog-2* or *baf-1(G12T)* males. Error bars represent standard deviation. *P* values from two-sided t-test are indicated; *p* values ≥ 0.05 are considered not significant (n.s.).

© The Author(s)

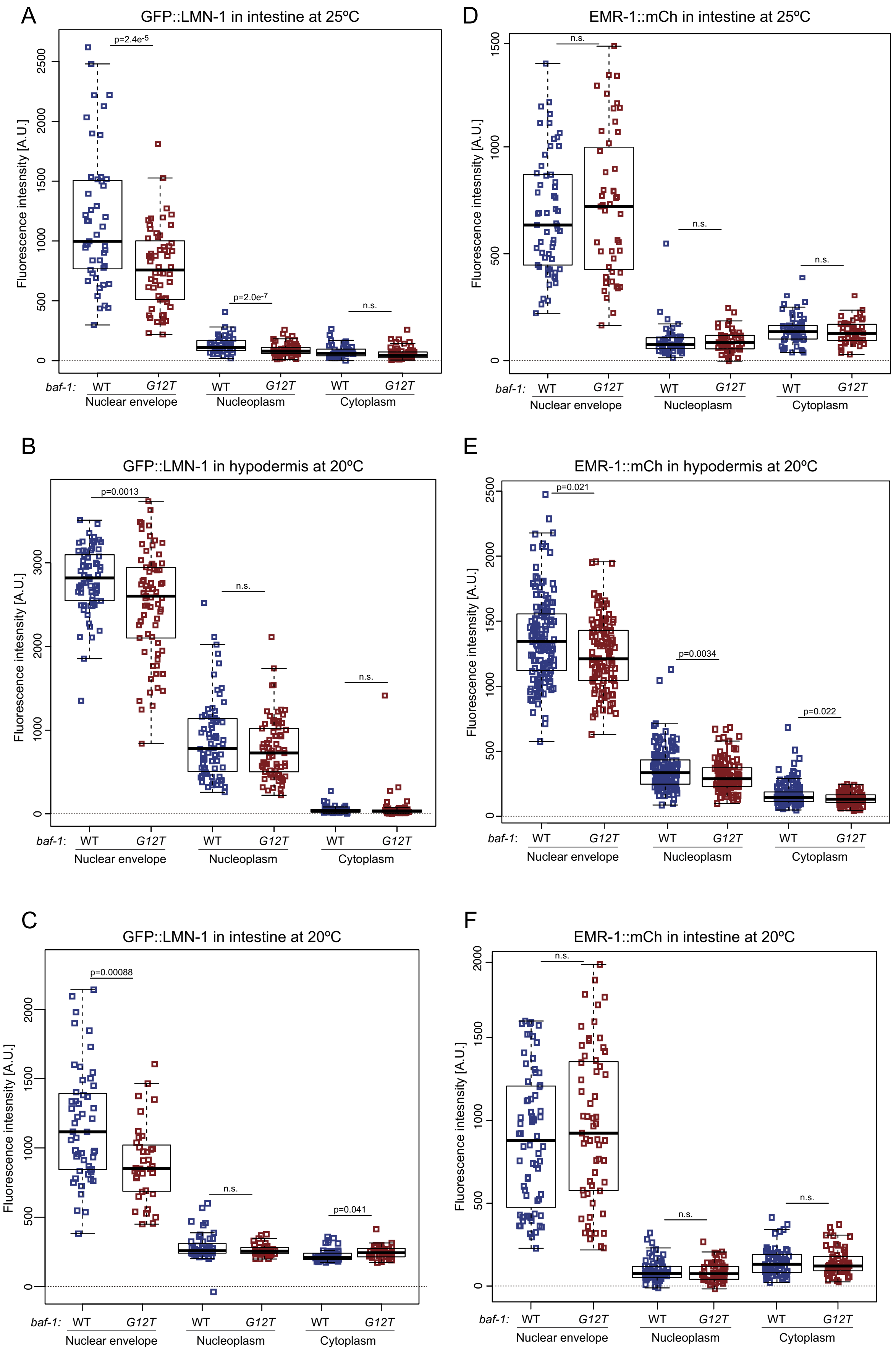
A
GFP::LMN-1 in intestine at 25ºC
Fluorescence intesnsity [A.U.]
p=2.4e-5
p=2.0e-7
n.s.
baf-1: WT G12T WT G12T WT G12T
Nuclear envelope Nucleoplasm Cytoplasm
B
GFP::LMN-1 in hypodermis at 20ºC
Fluorescence intesnsity [A.U.]
p=0.0013
n.s.
n.s.
baf-1: WT G12T WT G12T WT G12T
Nuclear envelope Nucleoplasm Cytoplasm
C
GFP::LMN-1 in intestine at 20ºC
Fluorescence intesnsity [A.U.]
p=0.00088
n.s.
p=0.041
baf-1: WT G12T WT G12T WT G12T
Nuclear envelope Nucleoplasm Cytoplasm
D
EMR-1::mCh in intestine at 25ºC
Fluorescence intesnsity [A.U.]
n.s.
n.s.
n.s.
baf-1: WT G12T WT G12T WT G12T
Nuclear envelope Nucleoplasm Cytoplasm
E
EMR-1::mCh in hypodermis at 20ºC
Fluorescence intesnsity [A.U.]
p=0.021
p=0.0034
p=0.022
baf-1: WT G12T WT G12T WT G12T
Nuclear envelope Nucleoplasm Cytoplasm
F
EMR-1::mCh in intestine at 20ºC
Fluorescence intesnsity [A.U.]
n.s.
n.s.
n.s.
baf-1: WT G12T WT G12T WT G12T
Nuclear envelope Nucleoplasm Cytoplasm

 © The Author(s)

Figure EV3. Nuclear envelope accumulation of LMN-1 is decreased in *baf-1(G12T)* mutants.

Quantification of GFP::LMN-1 (**A–C**) and EMR-1::mCh (**D–F**) signal in the NE, nucleoplasm and cytoplasm of intestinal and hypodermal cells at 20 °C and 25 °C as indicated. Data from 3 independent experiments; at least 15 animals were analyzed for each strain. *P* values from two-sided t-tests are indicated; *p* values ≥ 0.05 are considered not significant (n.s.).

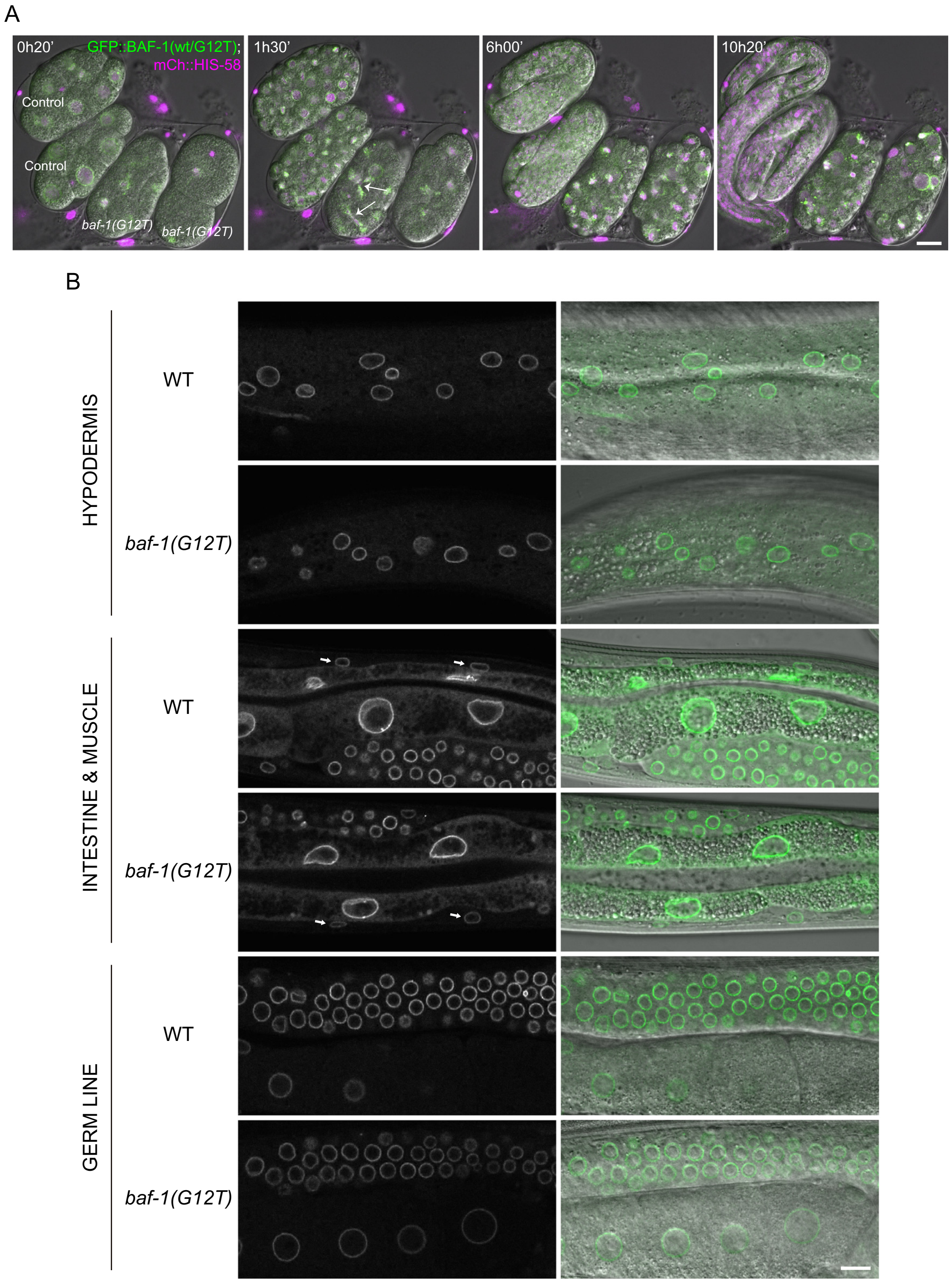

Figure EV4. ***baf-1(G12T)* worms are hypersensitive to GFP tagging.**

(**A**) Selected time points from confocal time-lapse recording of 2 GFP::BAF-1 and 2 GFP::BAF-1(G12T) endogenously tagged embryos co-expressing mCh::HIS-58 (magenta). Arrows indicate failures in chromosome segregation. (**B**) Confocal micrographs of hermaphrodites expressing endogenously tagged GFP::BAF-1 or GFP::BAF-1(G12T) as indicated. Scale bars represent 10 µm.

 © The Author(s)

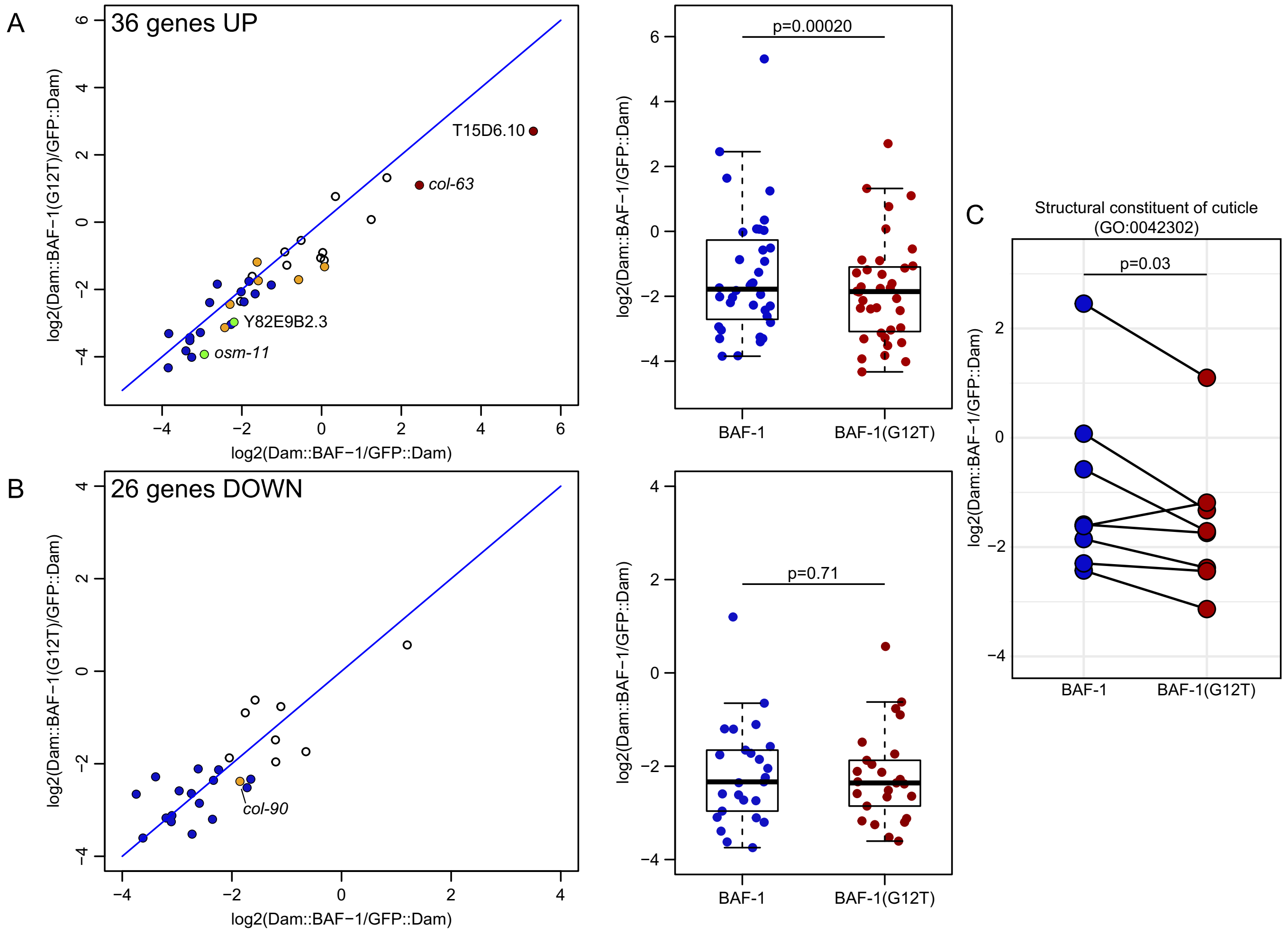

Figure EV5. Differentially expressed genes encoding cuticle components bind distinctively to BAF-1 and BAF-1(G12T).

(**A**, **B**) Genes with increased (**A**) or decreased (**B**) expression in hypodermis of *baf-1(G12T)* mutants were analyzed for association to wild-type BAF-1 (x axis) and BAF-1(G12T) in scatter plots (left; BAF-1 association on x axis; BAF-1(G12T) association on y axis) and boxplots (right). Genes with significantly higher association to Dam::BAF-1 or Dam::BAF-1(G12T) than to GFP::Dam are indicated in red in scatter plots. Genes indicated in orange (and *col-63* indicated in red) encode structural constituents of the cuticle. Genes plotted in green are also deregulated in intestine (see Fig. 6). (**C**) The association of differentially expressed genes encoding structural constituents of the cuticle to BAF-1 and BAF-1(G12T) in hypodermis. *P* values from two-sided paired t-tests, excluding outliers in (**A**, **B**), are indicated.

© The Author(s)
